# Evaluation of Physicians’ Awareness of Pediatric Diseases in Iran

**Published:** 2014-01-28

**Authors:** Hassan Abolhassani, Babak Mirminachi, Maedeh Daryabeigi, Zahra Agharahimi, Asghar Aghamohammadi, Ali Rabbani, Nima Rezaei

**Affiliations:** 1 Research Center for Immunodeficiencies; 2 Division of Clinical Immunology, Department of Laboratory Medicine, Karolinska Institute at Karolinska University Hospital Huddinge, Stockholm, Sweden; 3 Growth and Development Research Center, Children’s Medical Center; 4 Molecular Immunology Research Center; and Department of Immunology, School of Medicine, Tehran University of Medical Sciences, Tehran, Iran

**Keywords:** Pediatrics, Health Status of Children, Physicians’ Knowledge

## Abstract

***Objective:*** Physicians’ awareness about pediatric health problems is very important in health system. This has not been investigated in Iran as yet. Therefore this study was conducted to characterize the knowledge of the Iranian physicians which has direct association with health status of children.

***Methods:*** One hundred and four physicians, mainly pediatric specialists (58.6%) working in the state hospitals (45.1%) were enrolled. They filled a valid and reliable questionnaire, containing 26 questions about basic and important pediatric issues before and after an educational pediatric program (EPP).

***Findings*** ***:*** Thirty nine (37.5%) physicians answered correctly more than 2/3 of all questions (passed the examination) before EPP, which increased to 42.3% after EEP. Subgroup analysis showed that the total scores of general practitioners (*P*=0.007) was significantly increased after the EPP. Moreover, physicians with shorter practicing time (*P*=0.006) and those with shorter time past graduation (*P*=0.01) had a significant improvement in their total scores after the program. The best scores of educational issues were documented in growth and development (16.0%; *P*=0.04), followed by dermatology (9.2%, *P*=0.04), urology (9.1%; *P*=0.04), and asthma and allergy (9.0%, *P*=0.04).

***Conclusion:*** This study revealed that there are gaps in the knowledge of professionals about the pediatric issues.

## Introduction

Children are the most important human resources of the world, while attention to their health status is one of the critical issues worldwide. More than a century ago, with increasing physicians’ awareness of this fact that children have health problems and specialized diseases that are different from those of adults, the necessity of separated pediatrics discipline arose^[^^1^^-^^3^^]^. Since that time, most medical schools around the globe established department of pediatrics and are training pediatricians in specialized hospitals^[^^4^^-^^6^^]^. Nowadays, pediatrics’ disciplines concern with all aspects of children’s lives, including their physical, mental and psychological growth and development^[^^7^^]^.

 According to the United Nations Population Fund (UNFPA) census report in 2011, 32.16% of Iranian population consists of children less than 15 years^[^^8^^]^. According to World Health Organization (WHO) report in 2005, children health indicators in Iran were as follows: the percentage of underweight children 5.3%; neonatal mortality rate 13.3 per 1000 live births, and infant mortality rate 14.7 per 1000 live births. Furthermore, under-5 mortality rate was 25.1 per 1000 live births in 2006 and crude birth rate per 1000 population was 17% in 2007^[^^9^^]^. Unfortunately, according to World Bank report published in 2012, under-5 mortality rate rose to 25.8 (per 1000) and infant mortality rate increased to 21.8 (per 1000) in 2010^[^^10^^]^. 

 Despite several governmental efforts to gradate the level of health condition of children during recent years through primary health care system, no study has been conducted on physicians’ awareness of pediatric issues in Iran. We decided to design this study to characterize the knowledge of the Iranian physicians which has direct association with health status of children. Validated questionnaires were completed twice by physicians from different parts of Iran during the 24^th^ International Congress of Pediatrics, October 2012 in Tehran.

## Subjects and Methods

Population of this study was pediatricians (specialists and subspecialists), pediatric residents and general practitioners from different parts of Iran who participated in the 24^th^ International Congress of Pediatrics, October 2012, in Tehran. Prior to data collection the study was approved in the local ethics committee of Tehran University of Medical Sciences. Demographic data, university certificate, duration of medical practice and place of medical practice of participants were evaluated. The survey was performed in two separate parts (before and after) of the educational pediatric program (EPP). EPPs were included as integral part of International Congress of Pediatrics; the most important continued medical education on pediatric field in Iran. To assess a score of awareness of physicians on 30 different pediatric issues, a prototype questionnaire was prepared based on the most important key practical points by a special academic member who educates related issues in the EPP. A pilot study was performed to make the questionnaire reliable and valid (Cronbach's alpha=0.80, kappa coefficient=0.79). The final version of questionnaire with 26 closed questions was prepared and EPP program organized to encompass all of these questions based on the role of 1 question = 2 hours education. Table 1 contains the titles and areas of this questions as well as the number of questions in each field. The questionnaires were completed by participants before and after EEP; answer to more than 75% of questions was the cut-off point for including the completed form into analysis.

 The overall score of each participant was computed by adding the correct answers to these 26 questions. Passing the examination was defined as answering more than 2/3 of the questions. 

 Statistical analysis was performed using a commercially available software package (SPSS Statistics 16.0, SPSS, Chicago, Illinois). Appropriate method was recruited to evaluate the significance of description in various groups of participation (based on the demographic data) and also to compare scores before and after EPP in these groups. One-sample Kolmogrov-Smirnov test estimated whether data were normally distributed. Independent or paired tests via parametric and nonparametric analyses were performed based on the findings of this evaluation. A *P.* value of 0.05 or less was considered statistically significant in our study.

## Findings

The participating physicians included 104 individuals; most of them were pediatric specialists (61 persons, 58.6%) working in the state hospitals (47 persons, 45.1%). The median length of their practice in medicine was 16.2±11.6 (range 1-43) years. Other demographic data of studied individuals are illustrated in Table 1. The mean total knowledge score before educational pediatric program (EPP) was 16.7±4.8; thirty nine physicians (37.5%) answered correctly more than two thirds of all questions (those passed the examination). 

**Table 1:** Comparison of awareness score in different groups of 104 Iranian physicians educated in the EPP

**Variable**	**Parameter**	**Numbers ** **(%)**	**Mean of ** **Δ** **scores (SD)**	**Post Hoc** ***P. *** **value**	***P*** **. value**
**Age group**	≤30 years old	10 (9.6)	0.12(0.03)	-	0.05*
	31-60 years old	81 (77.8)	0.31(0.05)	(>60; *P*=0.04)	
	≥60 years old	13 (12.5)	-2.4(0.01)	(30-60; *P*=0.04)	
**Gender**	Male	56 (53.8)	-0.14(0.02)	-	0.2
	Female	48 (46.2)	-0.63(0.02)	-	
**Duration after last graduation**	≤ 10years	36 (34.6)	0.7 (0.01)	(10-20; *P*=0.001) (>20; *P*=0.04)	0.01*
	10-20 years	44 (42.3)	-0.3 (0.03)	(<10; *P*=0.001)	
	≥ 20 years	24 (23.0)	0.4 (0.05)	(<10; *P*=0.04)	
**Duration of medical practice**	≤ 10years	39 (37.5)	0.4 (0.02)	(>20;* P*=0.006)	0.03*
	10-20 years	33 (31.7)	0.1 (0.06)	-	
	≥ 20 years	32 (30.7)	-1.1 (0.04)	(<20; *P*=0.006)	
**Place of medical practice**	Only in state hospital	47 (45.1)	0.3 (0.1)	-	0.1
	Only in private hospital	4 (3.8)	0.12 (0.07)	-	
	Only in private office	19 (18.2)	-1.0 (0.06)	-	
	Overlapped places	34 (32.6)	-0.8 (0.04)	-	
**University certificate**	General pediatricians	15(14.4)	0.8 (0.03)	(PS; *P*=0.02) (SS; *P*=0.005)	0.01*
	Pediatric specialist	61(58.6)	-0.2(0.04)	(GP; *P*=0.02)	
	Sub-specialists	13(12.5)	-0.4(0.06)	(GP; *P*=0.005)	
	Pediatric resident	15(14.4)	1.0 (0.08)	-	
**Being faculty member**	Yes	14(13.4)	-0.2(0.03)	-	0.06
	No	90(86.6)	-0.3(0.07)	-	

After EPP this score was reduced non-significantly to 16.5±7.4 (*P*=0.7), but the percentage of physicians who passed the examination changed to 42.3%. Subgroup analysis showed that the total scores of general practitioners (12.7±3.2 *vs *13.5±5.0; *P*=0.007) and pediatric residents were increased after the EPP (17.6±4.1 vs 18.6±5.2; *P*=0.3). Moreover, physicians with shorter practicing time (*P*=0.006) and those with shorter time past graduation (*P*=0.01) had significant improvement in their total scores after the program (Table 2). 

 The best scores of educated issues were documented in growth and development field (16.0%; *P*=0.04), followed by dermatology (9.2%, *P*=0.04), urology (9.1%; *P*=0.04), and asthma and allergy (9.0%, *P*=0.04).

 In contrast, EPP had negative effects on the level of knowledge in the field of infantology (-0.17%; *P*=0.1), immunology (-1.0%; *P*=0.2) and imaging (-0.08%; *P*=0.2); however none of these changes were meaningful.

 Nutrition, dermatology and pulmonology fields achieved the worst scores both before and after EPP. Moreover, the best scores were achieved in the field of nephrology and psychiatrics (Table 2).

## Discussion

Pediatric education in Iran was initiated just before construction of the Children’s Medical Center Hospital (CMCH) in Tehran by the late Prof. Hassan Ahari and Prof. Mohammad Gharib^[^^11^^]^. This group of physicians established the first children’s department around the country; training and research activities begun since 1941. Children's specialized training courses were initiated with the establishment of the first specialized hospital for children (CMCH) in Tehran 1969 and the first group of pediatricians graduated in 1971. Subspecialty training courses began at CMCH in the field of pediatric nephrology 1976^[^^12^^]^. Pediatric subspecialty fields that already exist in Iran are pediatric nephrology, pediatric infectious diseases, pediatric cardiology, pediatric gastroenterology, pediatric endocrinology, pediatric immunology, pediatric rheumatology, pediatric hematology-oncology, pediatric pulmonology, pediatric neurology, pediatric psychology, pediatric surgery and infantology^[^^13^^]^. 

**Table 2:** Before/after percentage of correct answers to the question regarding pediatric field educated physicians in the EPP gathering from 104 participants

** Field of ** **question(s)**	**No of ** **question(s)**	** Title**	**Area**	**% of correct ** **answers ** **before EPP**	**% of correct ** **answers after ** **EPP**	***P*** **-value**
**Infectious diseases **	3	Scarlet fever	Diagnosis	0.89	0.95	0.08
		Skin rash	Diagnosis			
		Cough	Diagnosis			
**Endocrine**	2	Hyperlipidemia	Diagnosis	0.63	0.571	0.3
		Pheochromocytoma	Treatment			
**Asthma and** **Allergy **	2	Anaphylaxis	Treatment	0.69	0.78	0.04
		Allergy	Diagnosis			
**Pulmonology**	2	Varicella complication	Diagnosis and treatment	0.32	0.32	0.5
		Snoring	Diagnosis			
**Rheumatology and toxicology**	2	Lead poisoning	Diagnosis	0.65	0.62	0.4
		Joint pain	Diagnosis			
**Growth and development**	2	Normal growth	Diagnosis	0.76	0.93	0.04
		Normal growth	Definition			
**Infant**	1	Normal growth	Diagnosis	0.69	0.52	0.1
**Nephrology**	1	Hypertension	Treatment	0.93	0.96	0.2
**GI system**	1	Upper GI bleeding	Follow up	0.69	0.73	0.2
**Neurology**	1	Seizure	Diagnosis	0.63	0.57	0.3
**Immunology**	1	Human immunodeficiency virus infection	Diagnosis	0.89	0.78	0.2
**Urology**	1	Hematuria	Diagnosis	0.59	0.68	0.04
**Hematology**	1	ALL	Treatment	0.33	0.32	0.5
**Dermatology**	1	Burning	Treatment	0.28	0.37	0.04
**Vaccination**	1	Tetanus diphtheria vaccine	Health	0.56	0.52	0.4
**Nutrition**	1	Complementary nutrition	Health	0.26	0.34	0.2
**Psychiatric**	1	Depression	History Taking	0.91	0.84	0.06
**Surgery**	1	Side effects	Diagnosis	0.74	0.78	0.1
**Imaging**	1	Cancer	Diagnosis	0.50	0.41	0.2

ALL: Acute lymphoblastic leukemia; GI: Gastrointestinal

 There is a significant relationship between the duration of time passed from establishment and education of each subspecialty and percent of correct answers in the current study. As mentioned before, pediatric nephrology is the first established subspecialty in Iran^[^^11^^,^^14^^,^^15^^]^ and achieved the best score both before and after EPP. The first ward of pediatric subspecialty which was established in Iran was pediatric nephrology in 1976 by Prof. Bodaghi in CMCH and the first course of pediatric nephrology fellowship training established in Shiraz University of Medical Sciences^[^^16^^]^. Infectious diseases was the third, and second field regarding the percent of corrected answers, before and after EPP. This field was established 1987 by Prof. Siadati as the second established subspecialty in the field of pediatrics in Iran^[^^17^^]^. Immunology is the third field regarding the percent of correct answers and its ward was launched in 1988 by Prof. Farhoudi, while sub-specialty fellowship has begun since 1993^[^^17^^]^. 

 The findings of this study showed nutrition, dermatology and pulmonology fields achieved the worst scores both before and after EPP. Nutrition plays an important role in normal growth and development of children ^[^^18^^]^. The growth period of infancy is very important regarding neurocognitive development. Nutrition and growth during the first 3 years of life has a substantial effect on adult stature and some major health outcomes^[^^19^^]^. Lack of getting sufficient dietary needs can lead to energy and nutrient deficiency and has adverse effect on growth and developmental process. In parallel to the risk of nutrient deficiency, the increasing prevalence of obesity among children with negative health effects such as cardiovascular disease is emerging^[^^20^^,^^21^^]^. So, nutrition has very important impacts on various aspects of children’s health and improvement. The knowledge of physicians on this field is a necessity to reduce burden of nutritional disorder both in developed and developing countries^[^^22^^]^. 

 Pediatric pulmonology is a newly established subspecialty which has been launched in 2009. Respiratory disorders are the most frequent cause of hospital admission in children. Pediatric pulmonary disorders have very different manifestations than the same diseases in older children^[^^23^^-^^25^^]^. Acute respiratory infections are the most common cause of death, especially in developing countries. Bulletin of WHO indicates that pneumonia causes 6% of all deaths in Iranian children, which indicates the requirement of joining pediatric pulmonologists to infectious specialists in this regard^[^^26^^,^^27^^]^. Asthma is a chronic condition with increasing and substantial prevalence. The prevalence of asthma among Iranian children varies from 1.26% to 11.6%^[^^28^^]^. 

 Subspecialty of pediatric dermatology has not yet been launched in Iran^[^^17^^]^. However, there is no consensus about establishment of this discipline. Pediatric dermatology includes important aspects such as neonatal dermatology, genetic and non-genetic syndromes, eczema, vascular tumors and malformations, pediatric drug delivery and preventive health care^[^^29^^]^. Pediatric dermatology has an important role in the early diagnosis of genetic skin disorders^[^^30^^]^. Also some tumors are confined to childhood such as the Spitz nevus, the juvenile xanthogranuloma, mastocytoma, and hemangioma^[^^31^^,^^32^^]^. Moreover, many diseases that also occur in adults such as atopic dermatitis, eczema herpeticatum, psoriasis, scabies, lice infestation, and phototoxic or irritant reactions have different presentation in pediatric age and need special diagnostic and therapeutic methods^[^^33^^]^. 

 Despite the fact that just short time has passed from the establishment of pediatric rheumatology (since 2009), the percent of correct answers is approximately high^[^^17^^]^. Amazingly, the level of knowledge in the fields of infantology, immunology and imaging decreased after the educational program, which necessitates the need to change contents of EPP or to increase the time devoting to these fields during future continuous medical education programs.

 The findings of this study showed significant impact of EPP in the special group of physicians; doctors of earlier graduation and those with less experience in individual practice. This fact can reflect the effect of renewing data in those who have more updated information. On the other hand, reverse effect of EPP on elder physicians may indicate the alarm about the rigidity of elder doctors for accepting new data during EPP^[^^34^^,^^36^^]^. One third of the physicians in USA are over 65, and many continue to work with competence into their 70s and beyond, which arose concern about cognitive ability or physical skills putting their patients at risk^[^^37^^,^^38^^]^. Although the profession of medicine has never really had an organized way to measure physician’s competency, continued medical education and evaluation may be necessary for all physicians as well as regularly reviewing their outcomes on cases^[^^39^^,^^40^^]^.

## Conclusion

This study revealed that there are gaps in the knowledge of professionals about the pediatric issues. The results of this study also showed the value of establishment of special subspecialty in pediatric field on the general awareness of physicians in a special field. Moreover, it can be suggested that some pediatric fields have no well-organized duration of education prior to graduation of physicians, which leads to ineffectiveness of EPP on their knowledge. To compensate this, we suggest increasing the time of these special issues both in the timetable of common residency program and also in continued educational program to fill this gap for absence of fundamental education.
